# Wernicke Encephalopathy: A case report

**DOI:** 10.1192/j.eurpsy.2022.1149

**Published:** 2022-09-01

**Authors:** M. Jiménez Cabañas, F. Ruiz Guerrero, A. Bermejo Pastor, F. Mayor Sanabria, M. Fernández Fariña, M.D. Saiz González

**Affiliations:** 1 Hospital Clínico San Carlos, Instituto De Psiquiatría Y Salud Mental, Madrid, Spain; 2 Hospital Universitario Marqués de Valdecillas, Institute Of Psychiatry, Santander, Spain

**Keywords:** Wernicke, Korsakoff, alcohol, thiamine

## Abstract

**Introduction:**

We report a case of a 56-year old woman with a history of depressive disorder between 2012 and 2017 achieving full remission after treatment with antidepressants and anxiolytics. In the year 2021 was presented to the emergency department manifesting alteration of behavioral patterns, ataxia, mental confusion and horizontal nystagmus. A chronic alcohol abuse was also discovered while interviewing. She also exhibited low mood, anterograde amnesia and confabulations that improved rapidly after following treatment with thiamine.

**Objectives:**

Reviewing clinical manifestations and treatment of Wernicke encephalopathy and the development of Korsakoff syndrome.

**Methods:**

We systematically reviewed articles using PubMed.

**Results:**

Wernicke encephalopathy is a well-known complication of thiamine deficiency, mostly associated with alcohol use disorder. Classically, the syndrome comprises changes in mental status, gait ataxia and ophthalmoplegia. However, the full triad has been described in only 10-17 % of cases, which in our the case is report. After the diagnosis was established and was treated with thiamine, a rapid improvement in the patient´s clinical status was observed. Cognitive impairment was later identified, taking into account the possibility of a Korsakoff syndrome characterized by irreversible brain damage and subsequent loss of anterograde memory. In our patient, this specific diagnosis was dismissed due to the clinical improvement after thiamine treatment.

**Conclusions:**

It is relevant to emphasize the importance of a precise diagnosis and treatment of patients with Wernicke Encephalopathy to avoid the development of a Korsakoff syndrome.

**Disclosure:**

No significant relationships.

